# Endogenous erythropoietin concentrations and association with retinopathy of prematurity and brain injury in preterm infants

**DOI:** 10.1371/journal.pone.0252655

**Published:** 2021-06-02

**Authors:** Nancy M. Fahim, Michael K. Georgieff, Lei Zhang, Scott Naisbitt, Raghavendra B. Rao, Terrie E. Inder

**Affiliations:** 1 Department of Pediatrics, Washington University in St. Louis, St. Louis, Missouri, United States of America; 2 Department of Pediatrics, University of Minnesota, Minneapolis, MN, United States of America; 3 Division of Biostatistics, School of Public Health, University of Minnesota, Minneapolis, MN, United States of America; 4 Independent Researcher, Minneapolis, MN, United States of America; 5 Departments of Pediatrics, Neurology and Radiology, Washington University in St. Louis, St. Louis, Missouri, United States of America; Western University, CANADA

## Abstract

**Background:**

Endogenous erythropoietin (EPO) concentrations vary widely in preterm infants and may be associated with perinatal risk factors and neurological outcomes. Erythropoietin is elevated in fetal hypoxia but is also a potential neuroprotectant.

**Methods:**

In a prospective study of 27 infants ≤ 30 weeks gestation, serum erythropoietin concentrations were measured during the first month of life, on day 1 and weeks 1, 2, and 4, and related to perinatal risk factors and outcomes including retinopathy of prematurity and cerebral injury evaluated near term-equivalent post menstrual age using magnetic resonance imaging with quantitative scoring.

**Results:**

Lower birth weight was associated with higher EPO concentrations throughout the first 2 weeks of life (r = -0.6, p < 0.01). Higher day 1 and week 1 EPO concentrations were associated with lower Apgar score at 1 minute (r = - 0.5) and 5 minutes (r = -0.7), respectively (p < 0.01). Higher day 1 EPO concentrations and 2-week area under the curve were associated with increased risk (p = 0.01) and severity (r = 0.5, p < 0.02) of retinopathy of prematurity. Higher EPO concentrations at 2 weeks were associated with increased total brain injury score (r = 0.5, p < 0.05).

**Conclusion:**

Elevated endogenous erythropoietin concentrations in the first two weeks of life are associated with lower birth weight and increased risk of adverse outcomes.

## Introduction

Preterm birth is a major public-health challenge. Although survival has improved over the past two decades, children born preterm are at increased risk for cerebral palsy and cognitive and neurobehavioral deficits including lowered IQ, autism spectrum disorders, attention deficit hyperactivity disorder, anxiety disorders, and learning disabilities [1, 2]. Erythropoietin (EPO), a hematopoietic growth factor, has gained significant interest as a potential neuroprotective agent in this population.

The biological role of EPO is complex and extends well beyond hematopoiesis; EPO and its receptor (EpoR) are expressed early in fetal development in the human brain [3] and EPO is robustly up-regulated in response to fetal hypoxia [4]. EPO is thought to be neuroprotective due to its anti-apoptotic, neurotrophic, antioxidant effects and its promotion of angiogenesis and neurogenesis (reviewed in [4, 5]). In experimental stroke models, endogenously produced EPO contributes to the reduction of ischemic cerebral injury in models of ischemic or hypoxic preconditioning [6, 7].

Independent of the beneficial effects of EPO, its concentration may also be a biomarker of stressful perinatal conditions. Among preterm newborns, elevated concentrations of endogenous EPO have been associated with elevated inflammation-related proteins [8] and with a higher risk of lower Mental and/or Psychomotor Development Indices and microcephaly at 2 years [9]. Extremely preterm children with sustained elevations in inflammation-related proteins and EPO during the first postnatal month were also more likely to have cognitive impairment at 10 years [10]. Additionally, early endogenous EPO concentrations may convey information about risks of bowel, pulmonary, and retinal diseases in the very premature infant [11]. In these situations, it is unlikely that elevated EPO concentrations are causing neuropathology, given what is known about EPO neurobiology. Rather, they may reflect the response of preterm infants to specific medical conditions that threaten neurodevelopment.

The commonest neuropathology documented in the very preterm infant occurs in the cerebral white matter, and along with other forms of brain injury, has been shown to be best evaluated using magnetic resonance imaging (MRI) [12]. Although prior studies of endogenous EPO in preterm infants have considered associations with inflammatory proteins, diseases of prematurity and cognitive impairment, none has assessed brain injury as a function of endogenous EPO status over time using MRI prior to NICU discharge.

The factors that regulate endogenous EPO production in the preterm infant, as well as the influence of perinatal exposures, are not fully understood. This study investigates the impact of perinatal risk factors on endogenous EPO concentrations during the first month of life in preterm infants and the potential correlations between EPO concentrations and outcomes, including retinopathy of prematurity (ROP) and brain injury using MRI at term-equivalent post menstrual age.

## Materials and methods

The Washington University Institutional Review Board approved this research, protocol 08–0233. Written consent was obtained.

### Enrollment

Prospective data were collected between June 2008 and March 2009 at St Louis Children’s Hospital neonatal intensive care unit (NICU) in Saint Louis, Missouri, USA (altitude 150m) after IRB approval. Mothers and fetuses were not living at high altitude (>2500m). All preterm infants with gestational age (GA) ≤ 30 weeks expected to survive > 72 hours (as determined by the attending neonatologist) were eligible for recruitment. Exclusion criteria included infants with chromosomal abnormalities, major congenital anomalies, hemolytic blood disorders and suspected or proven congenital infections (e.g., toxoplasmosis, rubella, cytomegalovirus and herpes simplex). Patients missing both cord and day 1 EPO concentration values were also excluded. During periods of active enrollment, parents or guardians of consecutive eligible infants were approached by the investigators or a trained NICU research nurse to obtain written informed consent as soon as possible after delivery. All infants were treated according to the accepted care standards of the NICU.

### Data collection

Perinatal data were collected by review of maternal and infant charts by the investigators and a NICU research nurse. Information on perinatal risk factors and neonatal morbidities was collected. Prenatal factors included sex, race, gestational age, intrauterine growth restriction (IUGR), pregnancy-induced hypertension (PIH), preterm labor, maternal illnesses or drugs, multiple birth and antenatal steroids. Postnatal factors included birth weight, birth weight Z-score (calculated per Fenton et al. [13]), mode of delivery, Apgar score, sepsis, necrotizing enterocolitis (NEC), ROP, chronic lung disease (CLD), intraventricular hemorrhage (IVH), and packed red blood cell (RBC) transfusions received.

Cranial ultrasound (US) was obtained according to standard clinical practice, which included a scan within the first 7–14 days of life to evaluate for intraventricular hemorrhage (IVH). Infants were scanned using MRI using a 3T Tim Trio system (Siemens, Erlangen, Germany) without sedation near term gestational age or prior to discharge from the NICU. MR images were evaluated using a modified scoring system to assess brain injury as previously described [14]. The system evaluates and scores cerebral white matter (WM), cortical grey matter, deep gray matter, and cerebellum. All of the qualitative and quantitative assessments of MR images were performed by a single neonatal neurologist experienced with assessment of clinical MR images.

Serial determinations of serum erythropoietin (EPO) concentrations during the first month of life were performed in all infants upon enrollment in the study. Blood draws were taken only as indicated by routine clinical care; no extra blood draws were allowed for assessment of EPO concentration alone. The initial sample (First EPO) was assessed from serum collected ≤ 24 hours from birth; cord serum EPO was used when no such sample was available. Subsequent EPO concentrations were assessed at weeks 1, 2 and 4 of life if serum was available. EPO concentrations were analyzed using a solid state chemiluminescent immunometric assay system (Immulite 2000, Siemens Medical Solutions Diagnostics, Los Angeles, CA) with a 5.8% coefficient of variation. Hemoglobin concentrations (Sysmex XE, Kobe, Japan) were also recorded when obtained during the same blood draw. No patient received exogenous EPO.

### Statistical analysis

Descriptive statistics are expressed as frequencies, percent or mean ± standard deviation (SD) with range and median, as appropriate for baseline characteristics, outcomes and potential predictors. Due to skewed distribution of EPO concentrations, log transformation was applied. All the EPO analyses were in natural log scale. The area under the curve (AUC) of Ln(EPO) was calculated using the trapezoidal rule for those having first EPO and two-week EPO values (n = 20). To examine the change of Ln(EPO) over time, linear mixed model was used to account for within subject correlation. Ln(EPO) was compared between groups defined by baseline characteristics and outcome using two sample T-test. Partial correlation coefficients were calculated to explore the effect of confounders. Associations between Ln(EPO) at each time point and patient’s baseline characteristics were assessed using R-square and by Spearman correlation coefficient due to the small sample size and distribution of data.

All analyses were carried out using the SAS system (v. 9.4; SAS Institute, Cary, NC, USA). All P-values are two-sided and <0.05 is considered statistically significant. No adjustments were made for multiple comparisons.

## Results

### Study population

Forty-nine (49) infants were eligible for the study; parents of 7 declined, 7 were not approached due to unavailability of study investigators or social concerns, 7 were missing First EPO concentrations, and 1 died unexpectedly prior to consent. Twenty-seven subjects, 14 females and 13 males, were included in the study. The demographics and clinical characteristics of the study population are presented in Table 1. Mean gestational age was 27.5 ± 1.5 weeks and birth weight was 1094 ± 238g. Interventions and outcomes through NICU discharge, together with injury scores by MRI at 37 ±2 (range 33–43) weeks post menstrual age, are also summarized in Table 1. Two subjects died, and 44% of subjects received RBC transfusions. The mean total brain injury score was 5.2 ± 1.9.

**Table 1:** Perinatal characteristics, interventions and outcomes.

Parameter	Subjects (N = 27)
*Perinatal Characteristics*	
Gestational Age (wks)	27.5±1.5 (24–30, 27.6)
Birthweight (g)	1094±238 (734–1490, 1110)
Birth weight Z score	0.5±0.7 (-1.2–2.6, 0.6)
Male sex	13 (48)
Caucasian race	14 (52)
Maternal Smoking	4 (15)
IUGR	1 (4)
PIH	8 (30)
Antenatal steroids	21 (78)
Chorioamnionitis	5 (19)
PROM	6 (22)
Preterm labor	18 (67)
Vaginal Delivery	8 (30)
Abnormal placental pathology	20 (77)
Apgar score 1min	4.0±2.4 (1–8, 4)
Apgar score 5min	5.8±2.1 (1–9,6)
Low Apgar (<5) at 5min	6 (22)
Early sepsis (≤72h)	5 (19)
*Interventions and Outcomes*	
Death	2 (7)
NEC	1 (4)
Late sepsis	10 (37)
ROP, any	6 (22)
ROP, severe (grade 3)	2 (8)
CLD	8 (31)
RBC Transfusion	12 (44)
IVH (ultrasound grade ≥3)	3 (11)
MRI (N = 24)	
Total Injury Score	5.2±1.9 (2–9, 5)
Biparietal Diameter (mm)	69.4±4.7 (60.7–81.6, 70.0)
Transcerebellar Diameter (mm)	47.3±4.6 (38.1–60.5, 46.9)
White matter injury score	3.2±1.2 (1–5, 3)
Cortical grey matter injury score	0.8±1.0 (0–3, 0)

Binary variables presented as n (%), continuous variables as mean±SD (range, median). Abbreviations: IUGR, intrauterine growth retardation; PIH, pregnancy induced hypertension (preeclampsia); PROM, premature rupture of membranes; NEC, necrotizing enterocolitis; ROP, retinopathy of prematurity; CLD, chronic lung disease; RBC, red blood cell; IVH, intraventricular hemorrhage; MRI, magnetic resonance imaging. MRI near term-equivalent post-menstrual age was available for 24 (90%) of subjects.

**Fig 1:**
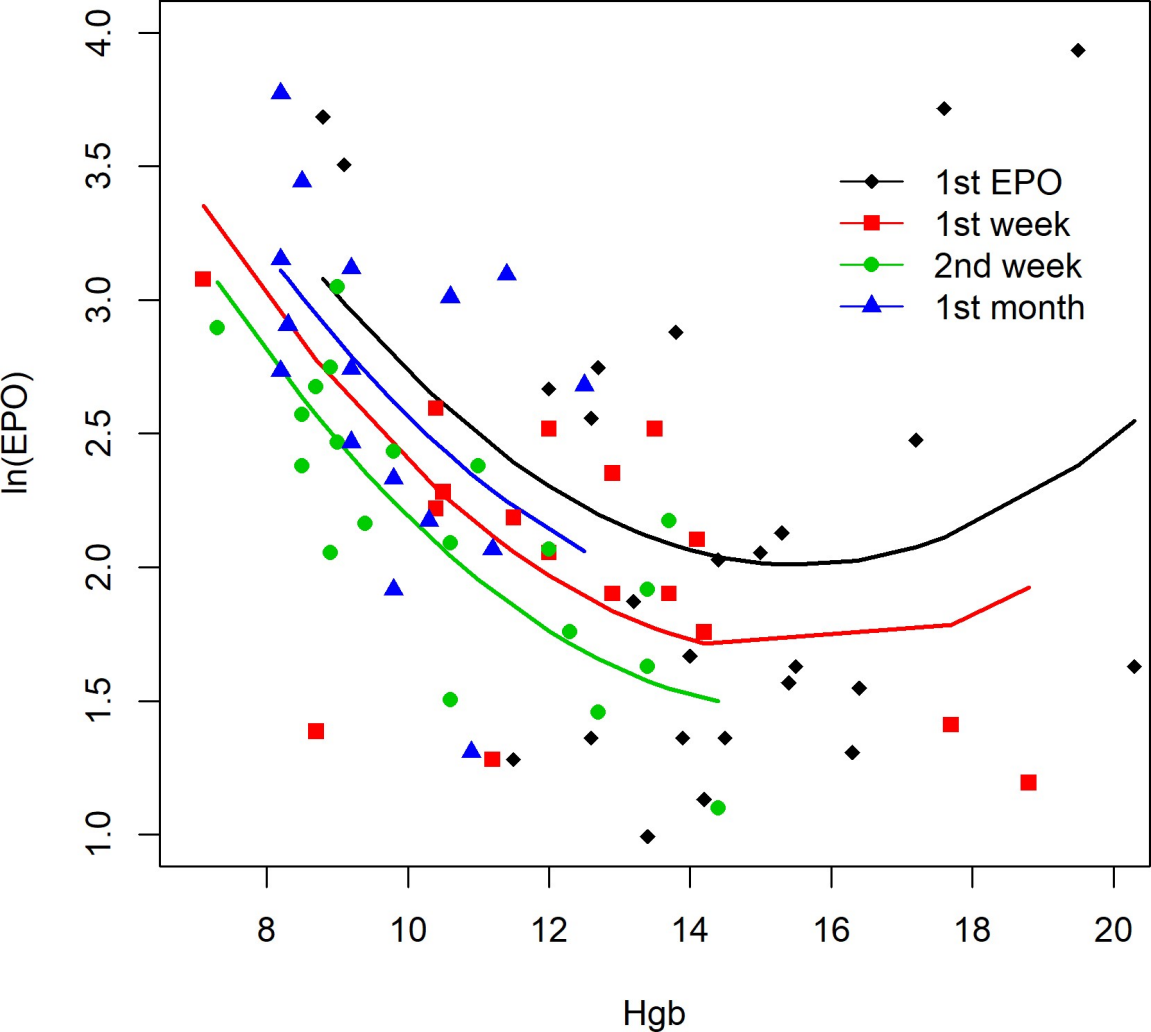
Association between Ln[EPO] and [Hgb] over time. Association between erythropoietin and hemoglobin concentrations over time. Scatterplot of ln[EPO] and [Hgb (g/dL)] at each time point. Fitted lines are from a linear mixed model (quadratic model).

First day EPO concentration [First EPO] showed wide variation with median 6.5 (range 2.7–174) mU/ml. Erythropoietin (EPO) and hemoglobin (Hgb) concentrations at each time point, and their interrelationship, are shown in Table 2 and Fig 1, respectively. EPO concentrations were not normally distributed; an outlier subject with very high EPO concentration had premature rupture of membranes (PROM), clinical chorioamnionitis, early sepsis, and anemia requiring RBC transfusions on day 2 and 7.

**Table 2:** Hemoglobin and erythropoietin concentrations over time.

Sample Time	Hgb (g/dL)	EPO (mU/mL)	ln[EPO]
Cord [n = 14]	NA	11.7±12.7 (2.9–51.1, 7.3)	2.1±0.8 (1.1–3.9, 2.0)
First* [n = 27]	14.2±2.7 (8.8–20.3, 14.0)	18.4±33.8 (2.7–174, 6.5)	2.2±1.0 (1.0–5.2, 1.9)
Week 1 [n = 17]	11.7±2.9 (7.1–18.8, 11.4)	8.7±4.6 (3.3–21.7, 8.2)	2.0±0.5 (1.2–3.1, 2.1)
Week 2 [n = 20]	10.7±2.0 (7.3–14.4, 10.6)	9.9±4.8 (3.0–21.1, 8.8)	2.2±0.5 (1.1–3.1, 2.2)
Week 4 [n = 17]	9.5±1.7 (7.3–14.0, 9.2)	17.1±9.8 (3.7–43.5, 15.4)	2.7±0.6 (1.3–3.8, 2.7)

Hemoglobin (Hgb) and Erythropoietin (EPO) levels are presented as mean±SD (range, median), together with the natural logarithm of EPO concentration (ln[EPO]) at each time point.

*First EPO defined as the first concentration taken <24 hours after birth (n = 25) with the additional inclusion of cord blood EPO for n = 2 subjects missing serum EPO on day 1.

### Association of EPO with perinatal variables

Lower birth weight was associated with higher EPO concentrations at each time point throughout the first 2 weeks of life, and birth weight negatively correlated with AUC_[EPO]0-2weeks_ (r = -0.6, p < 0.01) (Table 3). EPO concentrations at 1 week also negatively correlated with GA (r = -0.6, p < 0.02) (Table 3). After adjusting for GA, EPO concentrations remained negatively correlated with birth weight (r = -0.51, p = 0.025 at 2 weeks, and r = -0.47, p = 0.042 for AUC_0-2 weeks_) (S1 Table). EPO concentrations also negatively correlated with birth weight Z-score at First EPO (r = -0.39, p = 0.047) and at 2 weeks (r = -0.50, p = 0.025) (Table 3).

**Table 3:** Spearman correlation of ln[EPO] with risk factors and outcomes.

	ln(1^st^ EPO)		ln(1wk EPO)		ln(2wk EPO)		ln(1mo EPO)		ln(AUC 0-2wk EPO)	
Variable	r	p	r	p	r	p	r	p	r	p
Gestational age	-0.283	0.153	-0.581	**0.015**	-0.345	0.137	0.245	0.343	-0.375	0.104
Birth weight	-0.437	**0.023**	-0.555	**0.021**	-0.590	**0.006**	0.254	0.326	-0.575	**0.008**
Birth weight Z score	-0.385	**0.047**	-0.141	0.589	-0.501	**0.025**	0.174	0.504	-0.438	0.054
Apgar at 1 min	-0.506	**0.008**	-0.536	**0.032**	0.199	0.415	0.077	0.777	-0.383	0.106
Apgar at 5 min	-0.344	0.086	-0.680	**0.004**	0.120	0.624	0.202	0.453	-0.309	0.199
ROP stage	0.520	**0.008**	0.422	0.103	0.291	0.227	-0.322	0.225	0.535	**0.018**
IVH grade	0.341	0.082	0.373	0.141	-0.028	0.906	-0.195	0.453	0.376	0.102
RBC Transfusions (number of)	0.560	**0.002**	0.684	**0.003**	0.450	**0.046**	-0.414	0.098	0.590	**0.006**
Hemoglobin										
Day 1	-0.146	0.467	-0.358	0.158	0.004	0.987	0.372	0.142	-0.192	0.416
Week 1	-0.183	0.414	-0.445	0.074	-0.409	0.103	0.411	0.114	-0.409	0.103
Week 2	-0.136	0.507	-0.274	0.288	-0.743	**0.0002**	0.312	0.223	-0.304	0.192
Week 4	-0.117	0.604	-0.131	0.628	-0.472	0.056	-0.531	0.034	-0.453	0.068
MRI (~40wk GA)										
Total Brain Injury Score	0.123	0.566	0.124	0.648	0.477	**0.045**	-0.281	0.291	0.170	0.500
Biparietal diameter	-0.299	0.156	-0.222	0.409	-0.269	0.280	-0.032	0.905	-0.356	0.147
Transcerebellar diameter	-0.065	0.764	-0.207	0.442	-0.002	0.995	-0.046	0.867	-0.131	0.604
White matter injury	0.191	0.372	0.002	0.995	0.398	0.102	-0.100	0.714	0.246	0.325
Grey matter injury	0.013	0.950	-0.089	0.743	0.424	0.079	0.079	0.771	-0.011	0.967

Spearman correlation coefficient estimate, p-value, and r are presented for association of ln(EPO) at each time point with continuous variables. Abbreviations: ROP, retinopathy of prematurity; IVH, intraventricular hemorrhage; MRI, magnetic resonance imaging; GA, gestational age.

Higher early EPO concentrations were associated with lower Apgar scores. First EPO negatively correlated with 1 minute Apgar score (r = -0.51, p = 0.008), and 1 week EPO negatively correlated with 5 minute Apgar score (r = -0.68, p = 0.004) (Table 3). These associations were preserved after adjusting for GA (S1 Table) or for birth weight Z-Score (S2 Table).

### Association of EPO with outcomes, interventions, and neuroimaging

Early EPO concentrations were higher for subjects with ROP. First EPO and AUC_[EPO]0–2 weeks_ were significantly higher for patients with any (grade 1–3) ROP than for patients without ROP (ln[First EPO] 3.2 vs. 1.9, p = 0.01 and ln[AUC_[EPO]0–2 weeks_] 3.8 vs. 2.8, p = 0.01) (Table 4). Week 1 EPO was significantly higher for severe (grade 3) vs. none or mild (grade 0–2) ROP (3.08 vs. 1.94, p = 0.026) (Table 4). First EPO concentration (r = 0.52, p = 0.008) and AUC_[EPO]0–2 weeks_ (r = 0.5, p = 0.018) also correlated with stage of ROP (Table 3). The positive correlation between First EPO concentration and stage of ROP was preserved after adjusting for GA or for birth weight Z-Score (S1 and S2 Tables).

**Table 4:** Association between ln[EPO] and binary risk factors, outcomes, and interventions.

	ln(1^st^ EPO)			ln(1wk EPO)			ln(2wk EPO)			ln (1mo EPO)			ln (AUC 0-2wk EPO)		
Variable	Yes	No	P value	Yes	No	P value	Yes	No	P value	Yes	No	P value	Yes	No	P value
Sex—Male	2.2(1.2)	2.3(1.0)	0.74	2.1(0.6)	2.0(0.5)	0.65	2.2(0.6)	2.2(0.5)	0.96	2.5(0.6)	2.9(0.6)	0.22	3.0(0.9)	3.2(0.6)	0.71
Race -Caucasian	2.1(1.1)	2.3(1.0)	0.60	2.0(0.6)	2.1(0.4)	0.58	2.0(0.5)	2.4(0.4)	0.09	2.7(0.4)	2.6(0.8)	0.80	3.0(0.9)	3.2(0.6)	0.72
Maternal Smoking	1.8(1.3)	2.3(1.0)	0.36	1.8(0.6)	2.1(0.5)	0.35	1.9(0.4)	2.3(0.5)	0.22	2.7(0.3)	2.7(0.7)	0.87	2.7(0.6)	3.2(0.8)	0.20
IUGR	3.7	2.2(1.0)	0.14	NA	2.0(0.5)	NA	2.4	2.2(0.5)	0.62	3.8	2.6(0.6)	0.06	4.0	3.1(0.7)	0.25
PIH	2.3(1.0)	2.2(1.1)	0.89	1.9(0.5)	2.1(0.5)	0.30	2.0(0.5)	2.3(0.5)	0.24	3.0(0.6)	2.5(0.6)	0.14	3.0(0.7)	3.1(0.8)	0.69
Antenatal Steroids	2.2(1.1)	2.2(0.9)	0.94	2.1(0.6)	1.9(0.4)	0.54	2.1(0.5)	2.5(0.6)	0.18	2.6(0.7)	2.8(0.4)	0.58	3.1(0.8)	3.1(0.6)	0.99
Chorioamnonitis	2.4(1.9)	2.2(0.8)	0.65	1.8(0.5)	2.1(0.5)	0.28	2.1(0.5)	2.2(0.5)	0.82	2.5(0.3)	2.8(0.7)	0.49	3.2(1.3)	3.1(0.6)	0.85
PROM	2.6(1.7)	2.1(0.8)	0.28	1.9(0.5)	2.1(0.5)	0.49	2.1(0.4)	2.2(0.6)	0.55	2.6(0.3)	2.7(0.7)	0.78	3.2(1.1)	3.1(0.6)	0.67
Preterm labor	2.2(1.1)	2.3(1.0)	0.76	2.1(0.5)	2.0(0.5)	0.62	2.3(0.5)	2.0(0.5)	0.25	2.6(0.4)	2.7(0.9)	0.76	3.1(0.8)	3.0(0.7)	0.76
Vaginal delivery	1.7(0.7)	2.5(1.1)	0.07	2.0(0.7)	2.1(0.4)	0.78	2.1(0.5)	2.2(0.5)	0.48	2.5(0.4)	2.8(0.7)	0.39	2.7(0.5)	3.3(0.8)	0.07
Placental Pathology	2.4(1.1)	1.9(0.9)	0.31	2.0(0.6)	2.1(0.4)	0.99	2.3(0.4)	2.0(0.8)	0.34	2.6(0.7)	3.0(0.2)	0.24	3.3(0.8)	2.9(0.5)	0.30
Low Apgar 5m	2.6(1.0)	2.1(1.1)	0.37	2.3(0.6)	1.9(0.5)	0.15	2.1(0.2)	2.2(0.6)	0.73	2.2(0.7)	2.9(0.5)	**0.02**	3.2(0.5)	3.1(0.8)	0.74
Early sepsis	2.8(1.6)	2.1(0.9)	0.14	2.3(0.04)	2.0(0.6)	0.56	2.2(0.5)	2.2(0.5)	0.98	2.5(0.3)	2.7(0.7)	0.64	3.6(1.1)	3.0(0.6)	0.12
Transfusion	2.7(1.0)	1.8(0.9)	**0.016**	2.5(0.3)	1.8(0.4)	**0.004**	2.4(0.4)	1.9(0.5)	**0.03**	2.3(0.7)	2.9(0.4)	**0.048**	3.5(0.6)	2.7(0.6)	**0.01**
ROP–severe (3)	2.7(0.2)	2.2(1.1)	0.50	3.1	1.9(0.4)	**0.026**	2.6(0.7)	2.1(0.5)	0.30	2.5(0.9)	2.8(0.5)	0.47	3.6(0.1)	3.0(0.8)	0.38
ROP–any (1,2,3)	3.2(1.1)	1.9(0.9)	**0.01**	2.7(0.6)	1.9(0.5)	0.057	2.5(0.4)	2.1(0.5)	0.16	2.5(0.5)	2.9(0.5)	0.18	3.8(0.8)	2.8(0.6)	**0.01**
CLD	2.8(1.1)	2.0(0.9)	0.06	2.2(0.3)	2.0(0.6)	0.48	2.4(0.4)	2.1(0.6)	0.29	2.4(0.7)	2.8(0.5)	0.20	3.5(0.9)	2.9(0.6)	0.11
IVH (severe)	2.6(0.9)	2.2(1.1)	0.46	2.2	2.0(0.5)	0.74	2.3(0.1)	2.2(0.5)	0.78	2.2	2.7(0.6)	0.40	3.3(0.1)	3.1(0.8)	0.65
Death	2.2(0.7)	2.2(1.1)	0.94	2.6	2.0(0.5)	0.29	2.2	2.2(0.5)	0.98	1.3	2.8(0.5)	**0.01**	3.2	3.1(0.8)	0.88

Ln(EPO) at each time point and area under the curve (AUC) of ln(EPO) from 0 to 2 weeks presented as mean(SD) with two-sample t-test comparing subjects with presence (yes) vs. absence (no) of each binary variable. Abbreviations: IUGR, intrauterine growth retardation; PIH, pregnancy induced hypertension (preeclampsia); PROM, premature rupture of membranes; ROP, retinopathy of prematurity; CLD, chronic lung disease; IVH, intraventricular hemorrhage.

**Fig 2:**
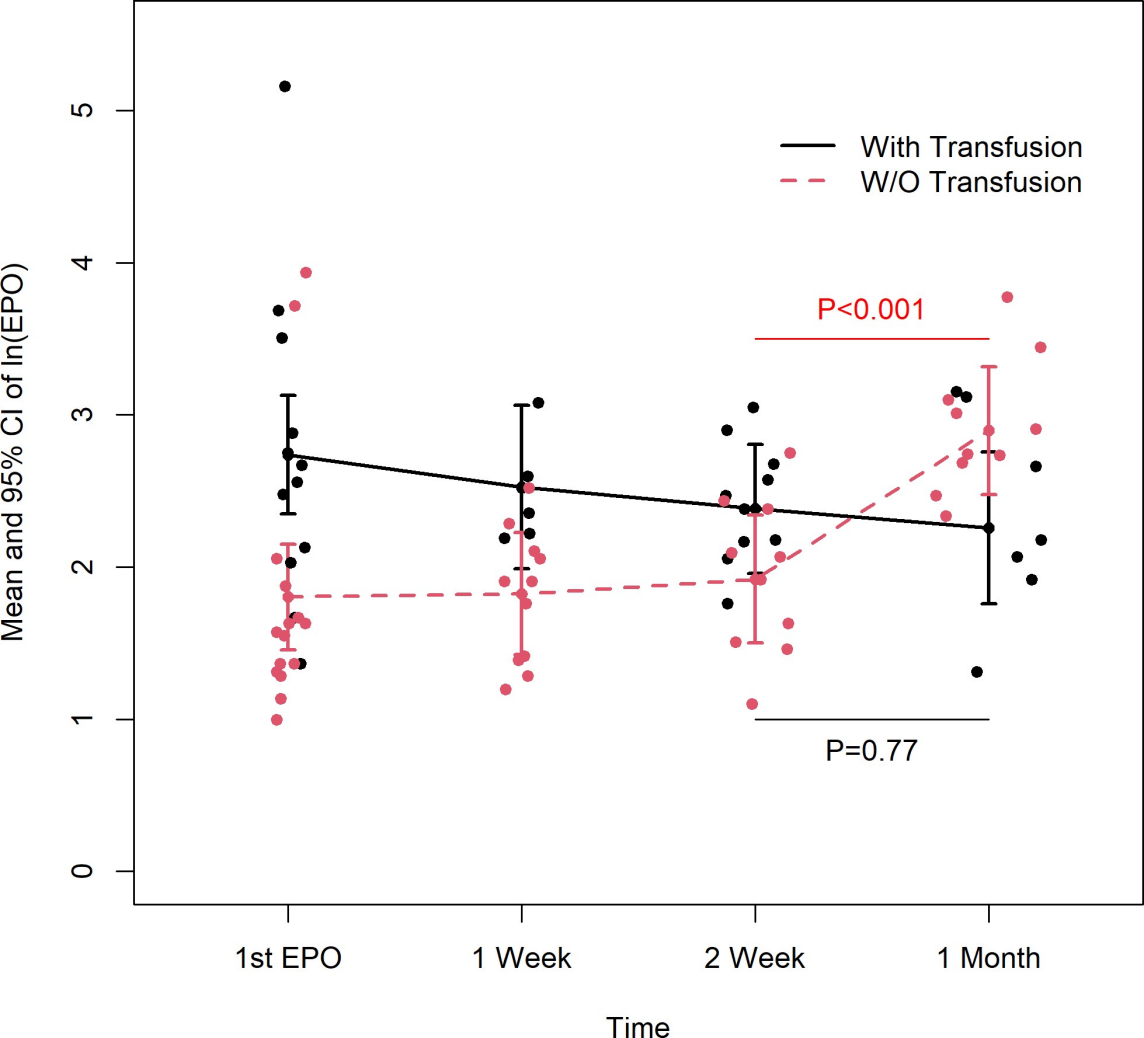
Ln[EPO] over time by RBC transfusion status. Ln[EPO] over Time by RBC Transfusion Status. Scatterplot of ln(EPO) at each time point for subjects with (black) and without (red) RBC transfusion, with mean and 95% confidence interval. First EPO was higher for subjects that required transfusion (p = 0.016); by 1 month, EPO was higher for non-transfused subjects (p = 0.048). [EPO] increased between week 2 and 4 only in the non-transfused group (p < 0.001 vs. p = 0.77).

A negative correlation between EPO and Hgb concentration was observed at 2 weeks (r = -0.74, p < 0.001) (Table 3) that was preserved after adjusting for gestational age (S1 Table) or birth weight Z-Score (S2 Table). Subjects receiving RBC transfusion had higher [First EPO] than those that did not (ln[First EPO] 2.7 vs. 1.8, p = 0.016) (Table 4 and Fig 2); this was also true at week 1 (ln[1wk EPO] 2.5 vs. 1.8, p = 0.004) and week 2 (ln[2wk EPO] 2.4 vs. 1.9, p = 0.029). In contrast, by month 1 the association was reversed, and EPO concentrations were instead lower in transfused vs. non-transfused patients (ln[1mo EPO] 2.3 vs. 2.9, p = 0.048). Endogenous EPO concentrations increased between week 2 and week 4 only in subjects that were not transfused (Ln[EPO] 1.9 to 2.9, p < 0.001), while concentrations remained unchanged during that period for transfused subjects (ln[EPO] 2.4 to 2.3, p = 0.77) (Table 4 and Fig 2).

The number of transfusions received during NICU stay correlated with stage of ROP (r = 0.83 unadjusted, r = 0.75 adjusted for GA, r = 0.79 adjusted for birth weight Z-Score), all with p < 0.0001 (Table 5). The number of transfusions also correlated with increased white matter injury by MRI (r = 0.45, p = 0.027 unadjusted; r = 0.50, p = 0.015 adjusted for GA) (Table 5). In gender subgroups, females but not males with transfusion had higher total brain injury scores than those without (7.0 ±1.2 vs. 4.9 ±1.9, p = 0.048 for females; 4.8 ±2.1 vs. 4.6 ±1.7, p = 0.88 for males) (S3 Table).

**Table 5:** Spearman partial correlation of number of transfusions with ROP stage and MRI injury scores.

	Unadjusted		Adjusted for GA		Adjusted for Birthweight		Adjusted for Z-Score	
***No*. *of Transfusions vs*.**	**r**	**p**	**r**	**p**	**r**	**p**	**r**	**p**
ROP Stage	0.830	**<0.0001**	0.747	**<0.0001**	0.729	**<0.0001**	0.792	**<0.0001**
MRI (~40wk GA)								
Total Injury	0.391	0.059	0.263	0.226	0.137	0.533	0.292	0.177
White Matter Injury	0.450	**0.027**	0.499	**0.015**	0.316	0.142	0.332	0.122
Grey Matter Injury	0.115	0.592	-0.097	0.659	0.087	0.693	0.196	0.370

Spearman correlation and partial correlation coefficients for association of number of transfusions with ROP and brain injury scores by MRI at near term-equivalent post-menstrual age, adjusted for GA, birthweight, and birthweight Z-Score. Abbreviations: GA, gestational age; Z-Score, birth weight Z-Score; MRI, magnetic resonance imaging; ROP, retinopathy of prematurity.

MRI imaging was completed for 24 subjects at 37 ±2 (range 33–43, median 37) weeks post menstrual age. A positive correlation of 2 week EPO with total brain injury assessed by MRI was observed (r = 0.48, p = 0.045) (Table 3) but not after adjusting for GA (r = 0.40, p = 0.110) or birth weight Z-Score (r = 0.41, p = 0.106) (S1 and S2 Tables, respectively). In contrast, the trend towards correlation of 2 week EPO and grey matter injury (unadjusted r = 0.42, p = 0.079) (Table 3) was significant after adjusting for birth weight Z-Score (r = 0.52, p = 0.031) (S2 Table). Exploratory analyses did not show that EPO concentration predicts brain injury differently by gender.

## Discussion

Endogenous Erythropoietin (EPO) concentrations in premature infants may both be a biomarker for hypoxic stress and have an influence on the response to brain injury. This study investigated whether EPO concentrations in the first month of life were driven by individual perinatal risk factors and were associated with subsequent outcomes and brain injury as assessed by MRI prior to NICU discharge.

EPO concentrations varied widely at birth, with a decline in point estimates to a nadir at about 1 to 2 weeks of life and recovery by week 4. This initial decline in EPO is consistent with early studies [15]. EPO concentrations through the first 2 weeks of life were higher for lower birth weight and lower birth weight Z-score infants.

Subjects with higher EPO concentrations at birth and 1 week had increased risk and severity of ROP. Higher initial and 1 week EPO concentrations were also associated with lower Apgar scores, and there was a trend towards higher initial EPO in subjects with perinatal stressors such as emergent caesarian section (2.45 vs. 1.66, p = 0.07), IUGR (3.72 vs. 2.16, p = 0.14), and early sepsis (2.84 vs. 2.08, p = 0.14) (Table 4). First EPO also trended higher for subjects with chronic lung disease. These findings are consistent with those reported by the ELGAN study investigators, in which high endogenous EPO concentrations during the first 2 postnatal weeks were associated with an increased risk of necrotizing enterocolitis (NEC) requiring surgery, ROP requiring treatment, and moderate BPD [11].

We interpret these results to indicate that these associations of early elevations in EPO with adverse outcomes are due to increased EPO production in response to fetal/neonatal hypoxia. Early studies have shown that fetal hypoxemia stimulates erythropoiesis and that it is a very responsive system; plasma EPO peaked less than 12 hours after the onset of hypoxemia in late-gestation fetal sheep [16]. Emergent C-section delivery is more often than not for fetal distress, unlike infants delivered via elective C-section without labor who had lower EPO concentrations than gestationally matched vaginal delivery infants–consistent with the hypoxemia hypothesis [17, 18]. IUGR is often secondary to maternal hypertension or placental pathology and is commonly associated with chronic fetal hypoxia [19]. Additionally, the increased risk and severity of ROP associated with elevated endogenous EPO concentrations at birth and during the first 2 weeks observed in this study may be related to underlying hypoxia. Hypoxia increases HIF-1α, which increases VEGF, the primary driver of neovascularization in ROP [20].

Teramo et al [4] hypothesized that since only low circulating EPO concentrations are required for red cell production, even when fetal hemoglobin concentrations are extremely low, the marked increases of fetal EPO concentrations in response to tissue hypoxia occur to protect the brain and other vital organs and suggests a broader role for EPO under these circumstances [4]. In the present study, elevated EPO concentrations throughout the first 2 weeks of life, although apparently insufficient to fully protect the brain, are associated with stressors that cause hypoxia and with adverse outcomes related to hypoxia. Early elevations in EPO may therefore serve as a biomarker for hypoxic injury; i.e., higher endogenous EPO concentrations may reflect a greater degree of fetal and neonatal hypoxia and are therefore associated with adverse outcomes including ROP and brain injury.

At birth through the first 2 weeks of life, EPO concentrations also negatively correlated with Hgb concentrations and were higher in infants that received transfusion than those that did not. Furthermore, EPO concentrations increased between weeks 2 and 4 only in the non-transfused group and by week 4 were higher in the non-transfused group than in the transfused group. These findings are consistent with appropriate physiological regulation of EPO production in preterm infants.

The number of transfusions received during NICU stay correlated with increased white matter injury by MRI. A reasonable hypothesis is a “double hit” to white matter: the hypoxia of low Hgb (leading to the transfusion in the first place) and the inflammation associated with transfusion [21]. Increased total brain injury was observed for transfused female but not male subgroups in this study, a result consistent with prior studies in which preterm infants were randomized to either a liberal or a restrictive threshold for transfusion, and a liberal criteria for transfusions resulted in worse outcomes overall and particularly for females [21, 22]. Preterm infants who received transfusions using liberal guidelines in these studies were found to have a smaller brain volume and reduced cerebral white matter at 12 years follow up, and females in the liberal group had the most abnormalities. It is unclear why transfused females appear to have worse developmental outcomes, but greater pro-inflammatory responses in females to RBC transfusions has been proposed as a potential etiology [21].

The number of transfusions received during NICU stay also correlated with stage of ROP (r = 0.8, p < 0.0001 unadjusted or after adjusting for GA or birthweight Z-Score). This finding may be relevant to current controversies over transfusion thresholds. Although no significant differences in severe ROP were observed between liberal and restrictive transfusion groups in the PINT randomized trial [23], a recent retrospective study of 1635 infants found early transfusions were associated with a nearly four-fold increase in risk of severe ROP, independent of GA at birth [24], consistent with the findings of this study. Although it is possible that relative tissue hypoxia caused both EPO stimulation and risk of ROP, and that transfusion is merely a marker of low hemoglobin, the correlation between transfusion and risk of ROP remained significant after adjusting for [EPO] (r = 0.6, p = 0.014 at 1 week; r = 0.8, p < 0.001 for AUC_[EPO]0–2 weeks]_, S4 Table) or hemoglobin (r = 0.7, p = 0.002 at 1 week, r = 0.8, p < 0.001 at 2 weeks, S4 Table), which suggests the impact of transfusion on ROP is not solely driven by lower oxygen carrying capacity.

Regarding possible etiologies for the potential impact of transfusion on injury, the lower EPO at 1-month observed in RBC-transfused patients may translate into loss of a neuroprotective growth factor that attenuates inflammation [22]. Decreased EPO production is not unexpected after RBC transfusion, and the right shift of the oxygen dissociation curve associated with transfusion of adult verses fetal hemoglobin (with different oxygen affinity) might exacerbate that decrease [25]. In addition, both white matter injury and ROP are resultant—at least in part—from oxidant stress [26–28]. Transfusion elevates iron levels and indeed free iron [27]. Transfusion-induced free iron can result in catalysis of free radical production by the Fenton reaction. This enhanced production of free radicals cannot be mitigated by the immature antioxidant system that is present in the preterm infant [26–28].

This study is unique in that it investigated endogenous EPO concentrations throughout the first four weeks of life and subsequent brain injury as assessed by MRI with systematic scoring of grey/white matter injury near term gestation. EPO concentrations at 2 weeks were associated with increased total brain injury score (r = 0.48, p = 0.045). However, this association lost significance (r = 0.40, p = 0.11) after correcting for GA or birth weight Z-Score. In contrast, the correlation of 2 week EPO concentration with grey matter injury was present only after adjusting for birth weight Z-Score (r = 0.52, p = 0.03). In comparison to other studies, pre-discharge MRI findings of increased brain injury in association with elevated EPO levels at 2 weeks would be consistent with and could presage the long-term neurodevelopmental deficits reported by the ELGAN study investigators. In ELGAN study subjects, endogenous EPO concentrations at 2 weeks of life, regardless of intermittent or sustained systemic inflammation, were associated with low mental and/or psychomotor development indices and microcephaly at 2 years [9] and with cognitive impairment at 10 years [10].

These results showing endogenous EPO concentration as a biomarker for hypoxic injury have no bearing on the potential protective effects of exogenous recombinant human EPO (rhEPO). Exogenous rhEPO is neuroprotective in animal experiments [5], and has been shown to decrease transfusion volumes [29] and improve neurodevelopmental outcomes in preterm human infants [30–32]. However, results vary, and recent prospective double-blind studies of very preterm infants in Switzerland and the United States failed to demonstrate a benefit for rhEPO administration over placebo on neurodevelopmental outcomes at 2 years [33, 34].

Independent of the potential benefit of rhEPO, endogenous EPO elevations within the first 2 weeks correlated in this study with morbidities associated with hypoxic injury including ROP and brain injury. Of interest, this association was not observed for EPO concentrations at 1 month of age, and anecdotally the lowest 1-month EPO concentration was observed in a single subject with NEC who died on day of life 38.

This study has limitations, including its small sample size with resulting low statistical power, and no adjustment was made for multiple comparisons. It is also limited to analysis of clinically discarded blood samples, which restricts both the number and volumes available for analysis. Furthermore, as in any observational study, confounding variables may complicate interpretation of findings. The MRI scans were performed at a range of postmenstrual ages, although the presence of injury should not influence the later MRI definition of injury presence. Finally, longer term clinical outcome data were not collected.

## Conclusions

Endogenous Erythropoietin (EPO) concentrations in the first month of life vary widely in preterm infants and may both be a marker for perinatal hypoxic injury and affect the healing response. Elevated EPO concentrations in the first two weeks are associated with lower birth weight, increased risk of ROP, and are suggestive of higher brain injury scores by MRI that may presage long term neurodevelopmental outcomes. RBC transfusions were associated with increased risk and severity of ROP and white matter injury. Additional studies are warranted to further asses the risks of transfusion and the potential role of early endogenous EPO as a biomarker for hypoxic injury in preterm infants.

## Supporting information

S1 Table Spearman partial correlations of ln[EPO] with risk factors and outcomes, adjusted for gestational age. Spearman partial correlation coefficient estimate adjusted for gestational age; p-value, and r are presented for association of ln(EPO) at each time point with continuous variables.
(PDF)

S2 Table Spearman partial correlation of ln[EPO] with risk factors and outcomes, adjusted for birth weight Z-score. Spearman partial correlation coefficient estimate adjusted for birthweight Z-Score; p-value, and r are presented for association of ln(EPO) at each time point with continuous variables.
(PDF)

S3 Table Brain injury by transfusion status in gender subgroups. Injury scores by MRI at near term gestational age are presented as mean (SD) with two-sample t-test comparing subjects with transfusion (yes) vs. without transfusion (no) in gender subgroups.
(PDF)

S4 Table Spearman partial correlation of transfusions with ROP stage and MRI injury scores, adjusted for [EPO] or [Hemoglobin]. Spearman correlation and partial correlation coefficients for association of number of transfusions with ROP and brain injury scores by MRI at near term-equivalent post-menstrual age, adjusted for ln[EPO] or [Hg] at week 1 or 2; coefficient estimate, p-value and r are presented.
(PDF)

S5 Table Association between ln[EPO] and continuous risk factors and outcomes by linear regression. Linear regression correlating ln(EPO) over time with continuous variables; coefficient estimate, p-value and R-square are presented.
(PDF)

S6 Table Association between ln[EPO] over time and continuous risk factors and outcomes by linear regression, adjusted for gestational age. Linear regression correlating Ln(EPO) over time with continuous variables adjusted for gestational age; coefficient estimate, p-value and r are presented.
(PDF)

S7 Table Association between Ln[EPO] and continuous risk factors and outcomes by linear regression, adjusted for birth weight Z-score. Linear regression correlating Ln(EPO) over time with continuous variables adjusted for birthweight Z-Score; coefficient estimate, p-value and r are presented (r = Pearson partial correlation).
(PDF)
