# Supplementary material for: Endogenous erythropoietin concentrations and association with retinopathy of prematurity and brain injury in preterm infants
Source: PLoS One. 2021 Jun 2;16(6):e0252655. doi: 10.1371/journal.pone.0252655 (PMC8171927; doi:10.1371/journal.pone.0252655)
Supplement: S1 Table — Spearman partial correlation coefficient estimate adjusted for gestational age; p-value, and r are presented for association of ln(EPO) at each time point with continuous variables. (PDF) [file pone.0252655.s001.pdf]

S1 Table. Spearman Partial Correlations of ln[EPO] with Risk Factors and Outcomes, Adjusted for Gestational Age

	ln(1st EPO)		ln(1wk EPO)		ln(2wk EPO)		ln(1mo EPO)		ln (AUC 0-2wk EPO)	
**Variable**	**r**	**p**	**r**	**p**	**r**	**p**	**r**	**p**	**r**	**p**
Gestational age	-	-	-	-	-	-	-	-	-	-
Birth weight	-0.355	0.075	-0.266	0.319	-0.511	**0.025**	0.151	0.577	-0.470	**0.042**
Birth weight Z score	-0.398	**0.044**	-0.349	0.185	-0.539	**0.017**	0.229	0.394	-0.478	**0.039**
Apgar at 1 min	-0.520	**0.008**	-0.584	**0.022**	0.218	0.385	0.075	0.791	-0.404	0.097
Apgar at 5 min	-0.305	0.139	-0.656	**0.008**	0.148	0.559	0.191	0.496	-0.311	0.210
ROP stage	0.468	**0.021**	0.462	0.083	0.118	0.640	-0.313	0.256	0.428	0.076
IVH grade	0.288	0.153	0.254	0.343	-0.083	0.735	-0.194	0.471	0.352	0.139
Transfusions (number of)	0.528	**0.006**	0.586	**0.017**	0.320	0.182	-0.345	0.191	0.492	**0.032**
Hemoglobin										
Day 1	-0.034	0.867	-0.105	0.699	0.211	0.386	0.299	0.261	-0.011	0.965
Week 1	0.103	0.656	-0.171	0.525	-0.265	0.321	0.437	0.103	-0.131	0.628
Week 2	0.035	0.867	0.162	0.549	-0.702	**0.001**	0.219	0.414	-0.160	0.512
Week 4	-0.118	0.610	-0.016	0.955	-0.457	0.075	-0.587	**0.022**	-0.436	0.091
MRI (~40wk GA)										
Total Brain Injury Score	0.042	0.851	0.044	0.876	0.402	0.110	-0.270	0.331	0.054	0.837
Biparietal diameter	-0.229	0.293	-0.087	0.758	-0.252	0.329	-0.034	0.904	-0.347	0.173
Transcerebellar diameter	-0.051	0.818	-0.190	0.498	-0.000	0.999	-0.040	0.888	-0.140	0.592
White matter injury	0.164	0.454	0.144	0.609	0.366	0.149	-0.102	0.717	0.199	0.443
Grey matter injury	-0.069	0.755	-0.174	0.536	0.348	0.171	0.111	0.693	-0.142	0.586

Spearman partial correlation coefficient estimate adjusted for gestational age; p-value, and r are presented for association of ln(EPO) at each time point with continuous variables. Abbreviations: MRI, magnetic resonance imaging; GA, gestational age; IVH, intraventricular hemorrhage; ROP, retinopathy of prematurity.
