# Supplementary material for: Endogenous erythropoietin concentrations and association with retinopathy of prematurity and brain injury in preterm infants
Source: PLoS One. 2021 Jun 2;16(6):e0252655. doi: 10.1371/journal.pone.0252655 (PMC8171927; doi:10.1371/journal.pone.0252655)
Supplement: S2 Table — Spearman partial correlation coefficient estimate adjusted for birthweight Z-Score; p-value, and r are presented for association of ln(EPO) at each time point with continuous variables. (PDF) [file pone.0252655.s002.pdf]

S2 Table. Spearman Partial Correlation of ln[EPO] with Risk Factors and Outcomes, Adjusted for Birth Weight Z-Score

	ln(1st EPO)		ln(1wk EPO)		ln(2wk EPO)		ln(1mo EPO)		ln (AUC 0-2wk EPO)	
**Variable**	**r**	**p**	**r**	**p**	**r**	**p**	**r**	**p**	**r**	**p**
Gestational age	-0.301	0.135	-0.637	**0.008**	-0.406	0.085	0.286	0.284	-0.423	0.071
Birth weight	-0.288	0.154	-0.555	**0.026**	-0.393	0.096	0.189	0.484	-0.420	0.073
Birth weight Z score	-	-	-	-	-	-	-	-	-	-
Apgar at 1 min	-0.435	**0.030**	-0.507	0.054	0.431	0.075	0.037	0.895	-0.280	0.260
Apgar at 5 min	-0.260	0.210	-0.662	**0.007**	0.329	0.182	0.174	0.536	-0.195	0.437
ROP stage	0.416	**0.043**	0.389	0.152	0.134	0.597	-0.270	0.330	0.443	0.065
IVH grade	0.427	**0.030**	0.409	0.116	-0.029	0.905	-0.238	0.375	0.421	0.072
Transfusions (number of)	0.482	**0.013**	0.678	**0.004**	0.318	0.185	-0.384	0.142	0.506	**0.027**
Hemoglobin										
Day 1	-0.205	0.316	-0.382	0.145	-0.047	0.848	0.415	0.110	-0.258	0.286
Week 1	-0.105	0.652	-0.430	0.097	-0.296	0.266	0.330	0.230	-0.346	0.190
Week 2	-0.037	0.860	-0.260	0.331	-0.692	**0.001**	0.274	0.304	-0.168	0.491
Week 4	-0.166	0.471	-0.193	0.492	-0.531	**0.035**	-0.517	**0.049**	-0.484	0.057
MRI (~40wk GA)										
Total Brain Injury Score	-0.052	0.815	0.108	0.701	0.406	0.106	-0.244	0.381	0.038	0.884
Biparietal diameter	-0.209	0.339	-0.291	0.293	-0.230	0.375	-0.056	0.843	-0.329	0.198
Transcerebellar diameter	0.032	0.884	-0.240	0.388	0.030	0.909	-0.037	0.897	-0.115	0.661
White matter injury	-0.022	0.920	0.010	0.973	0.279	0.278	-0.035	0.903	0.083	0.751
Grey matter injury	0.099	0.654	-0.110	0.697	0.523	**0.031**	0.052	0.853	0.040	0.878

Spearman partial correlation coefficient estimate adjusted for birthweight Z-Score; p-value, and r are presented for association of ln(EPO) at each time point with continuous variables. Abbreviations: MRI, magnetic resonance imaging; GA, gestational age; IVH, intraventricular hemorrhage; ROP, retinopathy of prematurity.
