# Supplementary material for: Endogenous erythropoietin concentrations and association with retinopathy of prematurity and brain injury in preterm infants
Source: PLoS One. 2021 Jun 2;16(6):e0252655. doi: 10.1371/journal.pone.0252655 (PMC8171927; doi:10.1371/journal.pone.0252655)
Supplement: S3 Table — Injury scores by MRI at near term gestational age are presented as mean (SD) with two-sample t-test comparing subjects with transfusion (yes) vs. without transfusion (no) in gender subgroups. (PDF) [file pone.0252655.s003.pdf]

S3 Table. Brain Injury by Transfusion Status in Gender Subgroups

	**Males** (n=11)			**Females** (n=13)		
**Transfusion (any)**	**Yes** (n=4)	**No** (n=7)	**p-value**	**Yes** (n=5)	**No** (n=8)	**p-value**
MRI (~40wk GA)						
Total Injury	4.8 (2.1)	4.6 (1.7)	0.880	7.0 (1.2)	4.9 (1.9)	**0.048**
White Matter Injury	3.3 (1.7)	2.4 (0.8)	0.294	4.2 (0.8)	3.3 (1.3)	0.143
Grey Matter Injury	0.3 (0.5)	1.3 (1.4)	0.189	1.2 (1.1)	0.3 (0.5)	0.126

Injury scores by MRI at near term gestational age are presented as mean (SD) with two-sample t-test comparing subjects with transfusion (yes) vs. without transfusion (no) in gender subgroups. Abbreviations: GA, gestational age; MRI, magnetic resonance imaging. MRI near term-equivalent post-menstrual age was available for 24 (90%) of 27 subjects, 11 of 13 (85%) males and 13 of 14 females (93%).
