# Supplementary material for: Endogenous erythropoietin concentrations and association with retinopathy of prematurity and brain injury in preterm infants
Source: PLoS One. 2021 Jun 2;16(6):e0252655. doi: 10.1371/journal.pone.0252655 (PMC8171927; doi:10.1371/journal.pone.0252655)
Supplement: S4 Table — Spearman correlation and partial correlation coefficients for association of number of transfusions with ROP and brain injury scores by MRI at near term-equivalent post-menstrual age, adjusted for ln[EPO] or [Hg] at week 1 or 2; coefficient estimate, p-value and r are presented. (PDF) [file pone.0252655.s004.pdf]

**S4 Table. Spearman Partial Correlation of Transfusions with ROP Stage and MRI Injury Scores, Adjusted for [EPO] or [Hemoglobin]**

| | **Unadjusted** | | **Adjusted for ln[EPO]** | | | | | | **Adjusted for Hg** | | | |
|---|---|---|---|---|---|---|---|---|---|---|---|---|
| | | | **1 week** | | **2 week** | | **AUC$_{0\text{-}2wk}$** | | **1 week** | | **2 week** | |
| *No. of Transfusions vs.* | **r** | **p** | **r** | **p** | **r** | **p** | **r** | **p** | **r** | **p** | **r** | **p** |
| ROP Stage | 0.830 | **<0.0001** | 0.617 | **0.014** | 0.818 | **<.0001** | **0.751** | **0.0003** | 0.654 | **0.002** | **0.836** | **<.0001** |
| MRI (~40wk GA) | | | | | | | | | | | | |
| Total Injury | 0.391 | 0.059 | 0.125 | 0.658 | 0.220 | 0.395 | 0.361 | 0.154 | 0.170 | 0.486 | 0.349 | 0.103 |
| White Matter Injury | 0.450 | **0.027** | 0.191 | 0.496 | 0.262 | 0.310 | 0.319 | 0.212 | 0.397 | 0.093 | 0.469 | **0.024** |
| Grey Matter Injury | 0.115 | 0.592 | 0.104 | 0.713 | -0.041 | 0.877 | 0.203 | 0.436 | 0.137 | 0.577 | 0.032 | 0.885 |

Spearman correlation and partial correlation coefficients for association of number of transfusions with ROP and brain injury scores by MRI at near term-equivalent post-menstrual age, adjusted for ln[EPO] or [Hg] at week 1 or 2; coefficient estimate, p-value and r are presented. Abbreviations: Hg, hemoglobin; GA, gestational age; Z-Score, birth weight Z-Score; MRI, magnetic resonance imaging; ROP, retinopathy of prematurity.
