# Supplementary material for: Endogenous erythropoietin concentrations and association with retinopathy of prematurity and brain injury in preterm infants
Source: PLoS One. 2021 Jun 2;16(6):e0252655. doi: 10.1371/journal.pone.0252655 (PMC8171927; doi:10.1371/journal.pone.0252655)
Supplement: S5 Table — Linear regression correlating ln(EPO) over time with continuous variables; coefficient estimate, p-value and R-square are presented. (PDF) [file pone.0252655.s005.pdf]

S5 Table. Association between ln[EPO] and Continuous Risk Factors and Outcomes by Linear Regression

Variable	ln(1st EPO)			ln(1wk EPO)			ln(2wk EPO)			ln (1mo EPO)			ln (AUC 0-2wk EPO)		
	**Coef**	**p**	**R^2**	**Coef**	**p**	**R^2**	**Coef**	**p**	**R^2**	**Coef**	**P**	**R^2**	**Coef**	**P**	**R^2**
Gestational age	-0.150	0.262	0.050	-0.212	**0.018**	0.320	-0.131	0.093	0.149	0.176	0.067	0.207	-0.171	0.141	0.117
Birth weight	-0.002	**0.041**	0.157	-0.002	**0.008**	0.383	-0.001	**0.004**	0.373	0.001	0.253	0.086	-0.002	**0.004**	0.386
Birth weight Z score	-0.353	0.207	0.063	-0.039	0.849	0.003	-0.241	0.091	0.151	0.012	0.946	0.0003	-0.365	0.082	0.159
Apgar at 1 min	-0.190	**0.022**	0.200	-0.084	0.072	0.214	0.040	0.458	0.033	0.025	0.680	0.013	-0.088	0.248	0.078
Apgar at 5 min	-0.133	0.177	0.075	-0.125	**0.019**	0.333	0.033	0.558	0.021	0.107	0.102	0.180	-0.068	0.408	0.041
ROP Stage	0.404	0.089	0.121	0.379	**0.021**	0.327	0.168	0.171	0.107	-0.137	0.290	0.080	0.299	0.093	0.157
IVH grade	0.208	0.217	0.060	0.171	0.150	0.133	0.016	0.879	0.001	-0.200	0.159	0.128	0.128	0.406	0.039
Transfusions (number of)	0.233	0.054	0.141	0.273	**0.004**	0.435	0.140	0.051	0.196	-0.142	0.130	0.146	0.214	**0.041**	0.212
Hemoglobin															
Day 1	-0.088	0.244	0.054	-0.057	0.172	0.121	-0.008	0.857	0.002	0.078	0.116	0.156	-0.062	0.326	0.054
Week 1	-0.086	0.298	0.054	-0.098	**0.023**	0.300	-0.106	**0.029**	0.280	0.090	0.085	0.197	-0.133	0.063	0.212
Week 2	-0.088	0.417	0.028	-0.099	0.127	0.148	-0.188	**<0.001**	0.609	0.061	0.422	0.044	-0.155	0.054	0.191
Week 4	-0.077	0.520	0.021	-0.079	0.346	0.064	-0.163	0.064	0.211	-0.218	0.069	0.218	-0.204	**0.032**	0.272
MRI (~40wk GA)															
Total Brain Injury Score	0.013	0.913	0.001	0.028	0.690	0.012	0.114	0.075	0.185	-0.073	0.355	0.061	0.059	0.568	0.021
Biparietal diameter	-0.052	0.289	0.051	-0.015	0.616	0.019	-0.026	0.337	0.058	-0.022	0.430	0.045	-0.044	0.288	0.070
Transcerebellar diameter	-0.011	0.825	0.002	0.001	0.983	<0.001	-0.006	0.823	0.003	-0.006	0.818	0.004	-0.012	0.758	0.006
White matter injury	0.100	0.598	0.013	-0.016	0.900	0.001	0.180	0.120	0.144	-0.047	0.724	0.009	0.161	0.386	0.047
Grey matter injury	-0.177	0.427	0.029	-0.007	0.960	0.0002	0.168	0.169	0.115	-0.035	0.758	0.007	-0.094	0.631	0.015

Linear regression correlating Ln(EPO) over time with continuous variables; coefficient estimate, p-value and R-square are presented. Abbreviations: Abbreviations: MRI, magnetic resonance imaging; GA, gestational age; IVH, intraventricular hemorrhage; ROP, retinopathy of prematurity.
