# Supplementary material for: Endogenous erythropoietin concentrations and association with retinopathy of prematurity and brain injury in preterm infants
Source: PLoS One. 2021 Jun 2;16(6):e0252655. doi: 10.1371/journal.pone.0252655 (PMC8171927; doi:10.1371/journal.pone.0252655)
Supplement: S6 Table — Linear regression correlating Ln(EPO) over time with continuous variables adjusted for gestational age; coefficient estimate, p-value and r are presented. (PDF) [file pone.0252655.s006.pdf]

**S6 Table. Association between ln[EPO] and Continuous Risk Factors and Outcomes by Linear Regression, Adjusted for Gestational Age**

| Variable | ln(1st EPO) | | | ln(1wk EPO) | | | ln(2wk EPO) | | | ln (1mo EPO) | | | ln (AUC 0-2wk EPO) | | |
|---|---|---|---|---|---|---|---|---|---|---|---|---|---|---|---|
| | **Coef** | **r** | **p** | **Coef** | **r** | **p** | **Coef** | **r** | **p** | **Coef** | **r** | **p** | **Coef** | **r** | **p** |
| Gestational age | - | - | - | - | - | - | - | - | - | - | - | - | - | - | - |
| Birth weight | -0.002 | -0.352 | 0.078 | -0.001 | -0.353 | 0.180 | -0.001 | -0.513 | **0.025** | 0.000 | 0.022 | 0.937 | -0.002 | -0.560 | **0.013** |
| Birth weight Z score | -0.406 | -0.292 | 0.147 | -0.174 | -0.260 | 0.331 | -0.296 | -0.507 | **0.027** | 0.111 | 0.171 | 0.528 | -0.438 | -0.501 | **0.029** |
| Apgar at 1 min | -0.187 | -0.452 | **0.023** | -0.067 | -0.453 | 0.090 | 0.043 | 0.213 | 0.395 | 0.025 | 0.124 | 0.659 | -0.084 | -0.282 | 0.256 |
| Apgar at 5 min | -0.111 | -0.226 | 0.278 | -0.085 | -0.442 | 0.099 | 0.052 | 0.239 | 0.340 | 0.089 | 0.393 | 0.147 | -0.048 | -0.150 | 0.552 |
| ROP Stage | 0.351 | 0.258 | 0.224 | 0.326 | 0.574 | **0.025** | 0.050 | 0.090 | 0.723 | -0.126 | -0.225 | 0.421 | 0.201 | 0.236 | 0.346 |
| IVH grade | 0.169 | 0.197 | 0.334 | 0.122 | 0.310 | 0.243 | -0.004 | -0.011 | 0.965 | -0.206 | -0.413 | 0.112 | 0.103 | 0.167 | 0.495 |
| Transfusions (number of) | 0.238 | 0.309 | 0.124 | 0.218 | 0.598 | **0.015** | 0.104 | 0.289 | 0.230 | -0.073 | -0.187 | 0.489 | 0.184 | 0.341 | 0.153 |
| Hemoglobin | | | | | | | | | | | | | | | |
| Day 1 | -0.064 | -0.159 | 0.438 | -0.021 | -0.142 | 0.601 | 0.024 | 0.131 | 0.593 | 0.051 | 0.266 | 0.319 | -0.030 | -0.110 | 0.653 |
| Week 1 | -0.001 | -0.002 | 0.993 | -0.060 | -0.336 | 0.203 | -0.082 | -0.387 | 0.139 | 0.075 | 0.337 | 0.219 | -0.081 | -0.271 | 0.309 |
| Week 2 | -0.017 | -0.029 | 0.892 | -0.013 | -0.049 | 0.857 | -0.189 | -0.735 | **0.0003** | -0.018 | -0.057 | 0.835 | -0.125 | -0.326 | 0.173 |
| Week 4 | -0.082 | -0.156 | 0.500 | -0.063 | -0.227 | 0.417 | -0.156 | -0.463 | 0.071 | -0.227 | -0.548 | **0.034** | -0.041 | -0.521 | **0.038** |
| MRI (~40wk GA) | | | | | | | | | | | | | | | |
| Total Brain Injury Score | -0.032 | -0.056 | 0.799 | 0.010 | 0.046 | 0.869 | 0.080 | 0.321 | 0.210 | -0.066 | -0.223 | 0.425 | 0.005 | 0.012 | 0.965 |
| Biparietal diameter | -0.040 | -0.175 | 0.423 | -0.006 | -0.066 | 0.814 | -0.023 | -0.241 | 0.351 | -0.021 | -0.213 | 0.446 | -0.046 | -0.263 | 0.308 |
| Transcerebellar diameter | -0.012 | -0.054 | 0.806 | -0.002 | -0.031 | 0.914 | -0.006 | -0.074 | 0.778 | -0.004 | -0.044 | 0.877 | -0.013 | -0.092 | 0.725 |
| White matter injury | 0.080 | 0.094 | 0.671 | 0.057 | 0.138 | 0.624 | 0.147 | 0.346 | 0.173 | -0.052 | -0.108 | 0.702 | 0.117 | 0.169 | 0.517 |
| Grey matter injury | -0.253 | -0.246 | 0.258 | -0.077 | -0.178 | 0.526 | 0.117 | 0.259 | 0.315 | -0.017 | -0.039 | 0.890 | -0.179 | -0.243 | 0.348 |

Linear regression correlating Ln(EPO) over time with continuous variables adjusted for gestational age; coefficient estimate, p-value and r are presented. Abbreviations: MRI, magnetic resonance imaging; GA, gestational age; IVH, intraventricular hemorrhage; ROP, retinopathy of prematurity.
