# Supplementary material for: Endogenous erythropoietin concentrations and association with retinopathy of prematurity and brain injury in preterm infants
Source: PLoS One. 2021 Jun 2;16(6):e0252655. doi: 10.1371/journal.pone.0252655 (PMC8171927; doi:10.1371/journal.pone.0252655)
Supplement: S7 Table — Linear regression correlating Ln(EPO) over time with continuous variables adjusted for birthweight Z-Score; coefficient estimate, p-value and r are presented (r = Pearson partial correlation). (PDF) [file pone.0252655.s007.pdf]

S7 Table. Association between Ln[EPO] and Continuous Risk Factors and Outcomes by Linear Regression, Adjusted for Birth Weight Z-Score

Variable	ln(1st EPO)			ln(1wk EPO)			ln(2wk EPO)			ln (1mo EPO)			ln (AUC 0-2wk EPO)		
	Coef	r	p	Coef	r	p	Coef	r	p	Coef	r	p	Coef	r	p
Gestational age	-0.177	-0.270	0.182	-0.235	-0.603	**0.013**	-0.161	-0.505	**0.027**	0.193	0.479	0.061	-0.215	-0.461	**0.047**
Birth weight	-0.002	-0.321	0.110	-0.002	-0.650	**0.006**	-0.001	-0.513	**0.025**	0.001	0.340	0.197	-0.002	-0.521	**0.022**
Birth weight Z score	-	-	-	-	-	-	-	-	-	-	-	-	-	-	-
Apgar at 1 min	-0.172	-0.411	**0.041**	-0.082	-0.451	0.091	0.065	0.311	0.209	0.027	0.118	0.676	-0.056	-0.191	0.449
Apgar at 5 min	-0.126	-0.270	0.193	-0.124	-0.580	**0.023**	0.040	0.183	0.467	0.107	0.425	0.114	-0.058	-0.192	0.446
ROP Stage	0.351	0.302	0.152	0.411	0.585	**0.022**	0.127	0.261	0.295	-0.137	-0.275	0.322	0.240	0.337	0.172
IVH grade	0.206	0.251	0.216	0.170	0.364	0.166	-0.002	-0.005	0.983	-0.200	-0.357	0.174	0.102	0.169	0.489
Transfusions (number of)	0.205	0.327	0.103	0.289	0.673	**0.004**	0.117	0.392	0.097	-0.146	-0.386	0.140	0.180	0.410	0.081
Hemoglobin															
Day 1	-0.112	-0.298	0.139	-0.063	-0.369	0.160	-0.022	-0.127	0.604	0.083	0.410	0.115	-0.085	-0.339	0.156
Week 1	-0.083	-0.218	0.342	-0.099	-0.548	**0.028**	-0.095	-0.462	0.072	0.073	0.365	0.181	-0.121	-0.403	0.122
Week 2	-0.057	-0.108	0.607	-0.100	-0.383	0.144	-0.176	-0.748	**0.0002**	0.064	0.211	0.432	-0.121	-0.349	0.143
Week 4	-0.088	-0.163	0.480	-0.094	-0.291	0.294	-0.171	-0.501	**0.048**	-0.223	-0.470	0.077	-0.211	-0.553	**0.026**
MRI (~40wk GA)															
Total Brain Injury Score	-0.058	-0.099	0.655	0.026	0.095	0.737	0.091	0.346	0.174	-0.075	-0.240	0.390	-0.002	-0.004	0.989
Biparietal diameter	-0.038	-0.168	0.442	-0.017	-0.150	0.595	-0.020	-0.196	0.450	-0.023	-0.228	0.414	-0.034	-0.219	0.399
Transcerebellar diameter	-0.007	-0.033	0.880	-0.000	-0.001	0.996	-0.004	-0.046	0.860	-0.006	-0.062	0.827	-0.010	-0.069	0.793
White matter injury	-0.032	-0.033	0.882	-0.025	-0.052	0.854	0.129	0.256	0.322	-0.040	-0.071	0.802	0.022	0.029	0.911
Grey matter injury	-0.133	-0.132	0.549	-0.010	-0.020	0.943	0.188	0.406	0.106	-0.040	-0.094	0.739	-0.061	-0.087	0.741

Linear regression correlating Ln(EPO) over time with continuous variables adjusted for birthweight Z-Score; coefficient estimate, p-value and r are presented (r = Pearson partial correlation). Abbreviations: MRI, magnetic resonance imaging; GA, gestational age; IVH, intraventricular hemorrhage; ROP, retinopathy of prematurity.
